# National

**Published:** 1896-05

**Authors:** 


*National.* Bulletin No. 11 of the Department of Agriculture gives some very important data and statistics relative to the dairy interests of the United States and the relative value of dairy products compared with other leading agricultural productions. Circulars 4 and 5 from the same department refer to the crossing of improved breeds of swine with the common hogs, or "razor-backs" of Florida, with a view of recovering some of the lost power and ability to resist the disastrous diseases of the western country. The second gives results of investigations into the infectious disease, designated entero-hepatitis in turkeys, which has proved so disastrous to the raisers of these birds in Rhode Island.

